# Abstract of the Proceedings of the Buffalo Medical Association

**Published:** 1865-04

**Authors:** Joseph A. Peters

**Affiliations:** Secretary


ART. II.—*Abstract of the Proceedings of the Buffalo Medical Association.*

TUESDAY EVENING, March 7, 1865.

The Association met pursuant to adjournment, the President in the Chair. Present Drs. Rochester, Miner, Strong, Gay, Congar, Hauenstein, Wetmore, Trowbridge, Cronyn, L. F. Harvey, Jansen, Johnson and Peters.

The minutes of the last meeting were read and approved.

*Dr. Rochester* said that the subjects of Pelvic Hæmatocele and Pyocele had often been presented to the Association, and he only brought them up at this time to relate a case, which was of interest simply because it opened spontaneously in an unusual place. The patient was a woman 27 years old, residing in the country, who was very ill at her confinement for two days and two nights, without medical attendance, and was finally delivered with instruments of a dead child. Her recovery was slow; she had vaginitis and a tumor formed in the left iliac region. An abscess formed between the uterus and bladder or the uterus and rectum, he could not say which, and pus was discharged. She was very ill, had no physician, and of course suffered a great deal. He, (Dr. R.) first saw the woman some four or five weeks ago, at which time she had a tumor in the hypogastric region, which he at first supposed to be the uterus containing menstrual fluid—particularly as on examination he could not detect an orifice in the *os uteri* large enough to admit a quill. The tumor finally opened spontaneously, externally, discharging pus, thus proving it to have been a pelvic pyocele, which was remarkable chiefly for its manner and place of opening.

*Dr. Miner* presented a specimen of bone from the skull of a soldier in hospital, including both tables, and presenting a bullet mark on the external table. It had been struck a glancing blow by the ball, and he supposed it to have been "*killed*" by the blow without any fracture having taken place. Thought such cases of death, and consequent sloughing of bone, though rare, did sometimes occur without actual fracture. This had been observed by military surgeons, and well authenticated cases reported.

This specimen of bone had been seen by several of the physicians of the hospital staff, and differences of opinion expressed as to the primary injury, the more general opinion being that its vitality had been destroyed by the shock, and that it had gradually separated as an exfoliation. When bone is deprived of its periosteal covering it is liable to exfoliate, but in many instances it will gradually heal, as if no such evil had occurred; it does not necessarily die or exfoliate. After injury of the scalp and removal of large patches of periosteum, it is common to see exfoliation of the outer table of the skull, or of a layer or scale of bone from its outer surface; this is productive of no great harm and only delays the recovery a little, unless the parts beneath are implicated. The specimen of bone involving both tables, is believed to be of interest, whatever might have been the primary lesion, since, if the explanation offered of its death, is not accepted, other points of interest still greater will be opened by supposing its separation the result of fracture.

*Dr. Rochester* related a case of a returned soldier who had erysipelas in front part of head, attended by severe pain and delirium. Two free incisions were made in the forehead, and a large amount of sanious pus was discharged. He went to Cincinnati, and he had since heard that a large portion of the frontal bone exfoliated, but could not say that both tables were involved. Would not any severe inflammation destroy bone?

*Dr. Strong* thought the pathology dubious. Thought the case was one of considerable, and, (if allowed as reported by Dr. M.,) one of extraordinary interest. But was its causation as contended for by the reporter, to be admitted as the true one? He must confess to having great doubt, or to speak more exactly, to having little doubt that it was not. He could see nothing in the case reported, to lift it out of the category of cases, in which the impact of the missile or weapon was sufficient to cause fracture, but not displacement. The lack of symptoms involving the brain and its meninges would point to this solution of the case, rather than to the extraordinary and well nigh *magical* cause given by the reporter. He thought it well, in either surgery or medicine, to remember the good rule in philosophy—to rest satisfied when we find a cause every way adequate to the effect, and not be tempted into the domains of the marvellous or the miraculous, for the purpose of elucidation. He believed it no uncommon thing in the annals of surgery that injury occurred to the skull sufficient to denude, or at least to impair the vitality of the periosteum, and to fracture the underlying bone through both its tables, but not sufficient to depress or displace the fractured portion, nor sufficient even to produce any considerable contusion externally. To his mind, this case (after examining the portion of parietal bone removed) seemed exactly to fit into the category of cases alluded to, and hence there was nothing either wonderful or very unusual in the case. With due deference to the surgical knowledge of the reporter, he must be allowed respectfully to demur, as aforesaid, to his view of the case.

*Dr. Wetmore*, after inquiring the name of the patient, remarked that he had treated him at the post hospital prior to Dr. Miner's seeing him, (that is in October last, soon after the wound was received,) for pneumonia, and at that time he had a great deal of suppuration from the wound, and a piece of bone as large as a twenty-five cent piece exposed. The fragment of bone was then movable though not displaced. Had in his possession two specimens much similar. Both were fractures.

*Dr. Miner* was glad of any direct testimony in a case it was impossible to decide without it; the case was increased in interest by any facts which could be substantiated concerning it. The soldier reports himself wounded October 19th, and furloughed in a few days for a visit to his friends; that his wound was only of the scalp, and produced no disturbance of the system at all; that he was not rendered unconscious at the time, but walked to the regimental hospital without assistance. He was admitted to the post hospital in Buffalo on account of inflammation of the lung, and not for the injury. If the bone was not displaced and was yet movable, it must have been a most wonderful instance of fracture, since at this early period there could scarcely have been absorption of adjacent bone to allow of motion. He was wounded October 19, and admitted to the General Hospital November 30th, a period intervening too short to admit of absorption of bone. Again that a ball should strike the skull obliquely and fracture from both its tables, a nearly circular piece, two inches in diameter, without other injury than a mark, where the ball struck, and without producing displacement or shock or after consequences of any sort, and still admitting of motion of the piece, will certainly constitute an aggregation of facts which are worthy of consideration, but which can hardly be said to "fit exactly into the category" of any rational belief. Yet since the data relied upon, appeared a little mixed, perhaps we should be unable to arrive at definite conclusions.

*Dr. Jansen* related a somewhat remarkable case of artificial opening in the trachea, but as he proposed presenting the subject of it to the Society at some subsequent meeting, a description of it is omitted here.

*Dr. Strong* reported a case of a child of G. S. B., female, weighing at birth some 2½ lbs. He was first called when it was three months old. Found it not thriving from nursing its mother, and advised partial weaning, and substituting cow's milk. Thereupon, after a few weeks, a change for the better was apparent. It continued to thrive finely, and, he saw little more of it till it reached 6½ months.

February 6th, was summoned in haste, the child being supposed to be dying. Found it had been left for a few minutes in its crib, (while the family was at breakfast,) feeling joyous and well. They soon noticed it became unusually still, and on going to it found its head fallen slightly forward on its chest, with respiration suspended, lips and face sub-livid, and quite unconscious. They immediately raised it up, blew in its face and mouth, and it soon resumed respiration and consciousness. She cried uncontrollably for an hour or so, but at length became quiet from a slight anodyne. Its bowels being somewhat inactive, he prescribed a few grains of calomel, and as the result it soon appeared as well and thriving as ever.

One week after he was again summoned in haste. Being near the residence he saw it within two or three minutes from the moment of seizure, but on arriving at its side it was dead, excepting one slight gasp.

He had detected no signs of cardiac disease, and as it was taken immediately out of town for burial, he could not obtain an examination; and, accordingly, was, and still is somewhat at a loss to account for its sudden death. He could only account for it by surmising, (what has always seemed to him somewhat mythical,) *spasm* of the *glottis*, or *epiglottis*, or both. He would be very glad to hear of any similar experience, or any exposition of the pathology of the case from gentlemen present.

*Dr. Congar* had seen a similar death in a case of scarlatina.

*Dr. Rochester* said death undoubtedly took place in the case narrated by Dr. Strong from spasm of the glottis. Such attacks, though rare, did occasionally occur, and were usually fatal. Was once called, in the absence of Dr. Eastman, to see one of that gentleman's patients, a little child, which had been seized in the way described by Dr. Strong. He snatched the little one up and commenced practicing "Marshal Hall's method," and with eventual success in saving its life, though it remained comatose for some time.

Prevailing diseases reported were, pneumonia, influenza, croup, laryngitis and pharyngitis, variola and varioloid.

*Dr. Rochester* called attention to the great extent to which variola was prevailing, not only in our city, but all over the country. He considered the prevalence of this disease a disgrace to a people, since means of prevention were so certain and easy of attainment. Thought the Association should take some action in its corporate capacity in the matter.

*Dr. Miner* called attention to the able and exhaustive prize essay on this subject, by Dr. Bell of Brooklyn, the suggestions of which had already been acted on in one or two cities, and ought to be here.

After some discussion by the gentlemen present it was resolved, on motion of Dr. Miner, that a committee of three, (to which the Health Physician, Dr. Eastman, was added,) be appointed to communicate the views of the Association to the Common Council through the Board of Health.

The President appointed as such Committee Drs. Miner, Rochester and Strong.

After which the Society adjourned.

JOSEPH A. PETERS, *Secretary.*

